# Growth Stunting Prevention in Indonesia: Dentist Knowledge and Perception

**DOI:** 10.1055/s-0042-1757465

**Published:** 2022-11-09

**Authors:** Zahira Mayfitriana, Anne Agustina Suwargiani, Arlette Suzy Setiawan

**Affiliations:** 1 Dentist Education Program, Faculty of Dentistry, Universitas Padjadjaran, Bandung, Indonesia; 2 Department of Community Dentistry, Faculty of Dentistry, Universitas Padjadjaran, Bandung, Indonesia; 3 Department of Pediatric Dentistry, Faculty of Dentistry, Universitas Padjadjaran, Bandung, Indonesia

**Keywords:** knowledge, perception, dentists, growth stunting, Indonesia

## Abstract

**Objective** Dentists in Indonesia as health workers have a role to play in improving the quality of life of others; thus, dentists' participation in growth stunting prevention is essential. It is also supported by the fact that growth stunting correlates with dental and oral health. Therefore, a dentist's knowledge and perception of growth stunting and its prevention affect the success of a dentist's role in the community.

This study aimed to explore the knowledge and perceptions of growth stunting and its prevention in dentists in Bandung.

**Materials and Methods** A descriptive study was conducted on general dental practitioners and dental specialists in Bandung. The number of participants gathered were 76 general dental practitioners and 30 dental specialists, which, if added all together, are 106 dentists as the study participants. Data was collected using a knowledge-based questionnaire containing three dimensions: knowledge about nutrition, growth and development, and health behavior. In addition, a perception questionnaire contains statements categorized into four dimensions: awareness, adoption, implementation, and maintenance. The questionnaire was distributed online using Google Form. A descriptive analysis was then done on the collected data.

**Results** Analysis of the findings showed that 80.19% of the participants have good knowledge of growth stunting and its prevention, 16.98% moderate, and 2.83% have poor knowledge. As for the perception category, the number of participants with positive perceptions is 53.77%, while the number of participants with negative perceptions is 46.23%.

**Conclusion** Most dentists in the city of Bandung have an excellent knowledge of growth stunting and its prevention and positively perceive growth stunting and its prevention.

## Introduction

Growth stunting has become a severe problem and cannot be ignored because many children under five in Indonesia still experience this disorder. Growth stunting is a condition where a child's growth is disrupted due to a lack of nutrition during a critical period of child development for an extended period. 1 The condition of malnutrition is one of the most important indicators of the welfare of a child's socioeconomic condition. In addition, growth stunting can cause health and mental development problems and reduce children's productivity and intellectual abilities. 2

Basic health research data from the Indonesia Ministry of Health in 2018 reveals that the national percentage of growth stunting is 30.8% which is more than what the World Health Organization (WHO) has put to the standard, which is 20%. 3 4 5 6 7 The data shows that Indonesia's growth stunting prevalence rate is still relatively high; thus, Indonesia is included in the top five countries where the growth stunting rate is still high compared to various countries in the world. 8 Therefore, reviewing the incidence of growth stunting in Indonesia is still a public health problem that must be the center of attention. 9

There is a relationship between growth stunting and oral health problems. Growth stunting can cause dental and oral health problems. 5 Furthermore, vice versa, dental and oral health problems can also cause growth stunting. 10 One of the dental and oral health problems that are very often found in children with growth stunting is dental caries. 11 Dental caries is a disease of dental tissue that begins with a demineralization process. Dental caries can interfere with the nutritional condition of children, and studies stated that dental caries in children could cause digestive disorders and difficulty eating. 12 13 Growth stunting can increase caries risk due to the reduced function of saliva as a buffer, cleanser, antisolvent, and oral antibacterial. 14 The impact of growth stunting in children can also be seen in the eruption of their teeth. 12 Growth stunting also has a relationship with oral health conditions in mothers and children through the influence of feeding behavior by mothers related to education and socioeconomic conditions of the family. 2 15

The high rate of growth stunting in Indonesia shows that preventive measures for growth stunting still need to be improved. Dentists in Indonesia as health workers have a role to play in improving the quality of life of others, so dentists need to participate in preventing growth stunting in Indonesia. It is also supported by the fact that growth stunting correlates with the oral health of mothers and children in Indonesia. 5 A dentist's knowledge and perception of growth stunting and its prevention affect the success of a dentist's role. Therefore, dentists need to have adequate knowledge and perception regarding this matter. Unfortunately, there has never been any research or data on the knowledge and perceptions of dentists regarding growth stunting and its prevention. Therefore, the purpose of this study is to determine the knowledge and perceptions of dentists in preventing growth stunting in dentists in the city of Bandung.

## Materials and Methods

This descriptive study has received ethical approval from the Research Ethics Commission of Padjadjaran with the number 994/UN6.KEP/EC/2021. This type of research is used to explain a phenomenon in the form of numbers describing a particular subject's characteristics. However, the technique used is a survey technique using a questionnaire instrument as a primary data collection tool.

### Study Participants

The population was general dental practitioner (GDP) dentists and dental specialists (DS) in the Bandung City area, totaling 1.488 based on data from the Indonesian Dental Association per September 2021. First, the minimum sample size was determined using the Slovin formula (confidence level of 95%, margin of error of 10%, population proportion of 60%) and led to a minimum of 94 samples. Then the sampling technique used is proportionate stratified random sampling, the technique of randomly taking samples from the population's members, stratified and proportional. This technique is used because the population of this study has members or elements that are not homogeneous and proportionally stratified. 16 The calculation results using the proportionate stratified random sampling formula (proportional stratified random sample) obtained a total of 76 samples for GDP and 30 for DS, a total of 106 dentists in Bandung.

### Questionnaire Development

The questionnaire in this study was divided into two parts; the first part consisted of questions regarding knowledge of growth stunting and its prevention, structured in the form of multiple-choice questions totaling 16 questions categorized into three dimensions: knowledge about nutrition, growth and development, and health behavior. Part two of the questionnaire contains questions about perceptions of growth stunting and its prevention in the form of statements and a scale of 1 to 5 (strongly disagree-strongly agree) that can be chosen by participants, totaling 20 questions that are categorized into four dimensions: awareness, adoption, implementation, and maintenance. This questionnaire has been tested for validity and reliability on 43 pediatric dentistry residents of Rumah Sakit Gigi dan Mulut Universitas Padjadjaran (Universitas Padjadjaran Dental Hospital) with a Cronbach Alpha value of 0.878.

### Data Analysis

The data that has been collected is analyzed descriptively. Then to get a descriptive discussion about the level of knowledge, the results are grouped according to the knowledge assessment criteria, namely, good knowledge if the value is 76 to 100%, sufficient knowledge if the value is 56-75%, and poor knowledge if the value is less than 56%. 17 18 Meanwhile, to get a descriptive discussion about the perception of growth stunting and its prevention, grouping is carried out according to the perception measurement criteria, which consists of the perception being declared positive if the total score obtained by the respondent is more than total mean score and negative perception if the total score obtained from the respondent is less than total mean score. 19

## Results

The total number of participants who filled out the questionnaire was 109, with 106 valid participants and three invalid participants due to unsigned informed consent. Of the 106 valid participants, there were 76 general and 30 specialist dentists. Characteristics of participants in this study include gender, age, qualifications, formal education after the dentist profession, occupation, length of work, and dental practice. The results of the descriptive analysis of the characteristics of the participants can be seen in Table 1 .

**Table 1:** Characteristics of participants

Characteristics	*n*	%
**Gender**		
Male	21	19.81
Female	85	80.19
**Age**		
17–25 years	3	2.83
26–35 years	41	38.68
36–45 years	25	23.58
46–55 years	30	28.30
56–65 years	7	6.60
**Qualification**		
General practitioner dentist	76	71.70
Specialist dentist	30	28.30
**Highest formal education after obtaining dentist profession**		
Have not taken further education yet	35	33.02
Currently pursuing a residency program	38	35.85
Master/specialty program	28	26.42
Doctorate	5	4.72
**Occupation**		
Government official	35	33.02
Private sector/non-governmental	71	66.98
**Long practice as a dentist**		
<5 years	24	22.64
5–10 years	23	21.70
>10 years	59	55.66
**Dental practice office**		
Independent practice	28	25.42
Dental hospital, dental clinic	46	43.39
Hospital	32	30.19

Data on dentists' knowledge about growth stunting and its prevention has been processed and then presented based on true, false, and unsure answers per indicator. The knowledge questionnaire about growth stunting and its prevention in this study consisted of three indicators; nutrition, growth and development, and behavior. Table 2 shows that the indicator with the correct answers on this questionnaire is nutrition at 93.40%, the percentage of correct answers on the growth and development indicator is 88.36%, and the percentage of correct answers on the behavioral indicator is 85.85%.

**Table 2:** Results of knowledge questionnaire answers per indicator

Sl. no.	Indicator	Answer					
		True		False		Not sure	
		*n*	%	*n*	%	*n*	%
1	Nutrition	198	93.40	0	0.00	14	6.60
2	Growth and development	843	88.36	42	4.40	69	7.23
3	Health behavior	455	85.85	38	7.17	37	6.98

Table 3 shows the results of the answers to the questionnaire per question. The highest distribution of nutrition indicators is participants with correct answers to question items regarding the definition of growth stunting, 98.11%, and uncertain answers as much as 1.89%. There was no respondent with wrong answers on this indicator. The highest distribution of growth and development indicators is participants with correct answers to question item regarding child dental caries, as much as 98.11%, and to question item regarding the impact of dental caries on children's nutrition, as much as 97.17%. The lowest distribution is participants with incorrect answers to questions regarding the vulnerable period in toddlers, as much as 0.94%. The highest distribution of behavioral health indicators is participants with the correct answer to the question regarding poor parenting and the incidence of growth stunting, as much as 92.45%. The lowest distribution is participants with unsure answers to question items regarding poor parenting and the incidence of growth stunting, as much as 1.89%.

**Table 3:** Results of knowledge questionnaire answers per question

Indicator	True		False		Not sure	
	*n*	%	*n*	%	*n*	%
Nutrition						
1. Definition of growth stunting	104	98.11	0	0.00	2	1.89
15. Risk factors for growth stunting: the number of caries in primary teeth	94	88.68	0	0.00	12	11.32
Growth and development						
4. Impact of dental caries on children's nutrition	103	97.17	3	2.83	0	0.00
5. Impact of infection and caries	97	91.51	6	5.66	3	2.83
7. The vulnerable period in toddlers	91	85.85	1	0.94	14	13.21
8. Correlation of growth stunting	96	90.57	3	2.83	7	6.60
9. Children's dental caries and consumption disorders	104	98.11	0	0.00	2	1.89
10. Impact of growth stunting on potential	98	92.45	5	4.72	3	2.83
11. Developmental period of growth stunting	90	84.91	6	5.66	10	9.43
13. Risk factors for growth stunting: malnutrition	86	81.13	6	5.66	14	13.21
14. Risk factors for growth stunting: maternal oral health	78	73.58	12	11.32	16	15.09
Health behavior						
2. Steps to prevent growth stunting	93	87.74	7	6.60	6	5.66
3. Poor parenting and the incidence of growth stunting	98	92.45	6	5.66	2	1.89
6. Habit of washing hands	82	77.36	14	13.21	10	9.43
12. Time to prevent growth stunting	92	86.79	6	5.66	8	7.55
16. Risk factors for growth stunting: disease history	90	84.91	5	4.72	11	10.38

The level of knowledge of growth stunting and its prevention in the city of Bandung can be seen in Table 4 , which shows that most dentists in the city of Bandung have a good level of knowledge about growth stunting and its prevention which is 80.19%.

**Table 4:** The level of knowledge of dentists about growth stunting and its prevention in the city of Bandung

Level of knowledge	*n*	%
Good	85	80.19
Sufficient	18	16.98
Insufficient	3	2.83
Total	106	100.00

Dentists' perception data on growth stunting and its prevention are also processed and presented based on assessment indicators. For example, Table 5 explains that the indicator with the most strongly agreeing answers on the perception questionnaire is the awareness indicator of 63.52%, and the percentage of answers strongly agreeing with the adoption indicator is 60%. The percentage of answers strongly agreeing with the implementation indicator is 61.13% and the percentage of answers strongly agreeing with the maintenance indicator is 59.12%.

**Table 5:** Results of perception questionnaire answers per indicator

Indicator	Answer									
	Strongly agree		Agree		Neutral		Disagree		Strongly disagree	
	*n*	%	*n*	%	*n*	%	*n*	%	*n*	%
Awareness	404	63.52	210	33.02	16	2.52	6	0.94	0	0.00
Adoption	318	60.00	184	34.72	22	4.15	6	1.13	0	0.00
Implementation	195	61.13	116	36.36	6	1.88	2	0.63	0	0.00
Maintenance	376	59.12	231	36.32	27	4.25	2	0.31	0	0.00

The results of the answers to the questionnaire per item are presented in Table 6 . The highest distribution of the awareness indicator is the statement regarding dentists maintaining dental and oral health, with as much as 72.64% in the strongly agree answer, while the lowest distribution, the strongly agree answer, is in the statement regarding the role of dentists in detecting growth stunting. The highest distribution on the adoption indicator is among participants who answered strongly agree with the item regarding dentists providing diet education, as much as 66.04%, and the lowest strongly agree answers are in the item regarding dentists contributing to antenatal care (ANC) as much as 54.72%. The highest distribution of the implementation indicators is participants who strongly agreed with the item regarding dentists' role in maintaining oral health for pregnant women, as much as 63.21%. However, the statement regarding dentists needing to play an active role in preventing growth stunting has the lowest strongly agree, as much as 58.49%. The highest distribution on the maintenance indicator is the respondent who answered strongly agree with the statement regarding active promotion of healthy food, as much as 66.04%, and for the distribution of answers strongly agree, the lowest is in a statement regarding dental and oral health education related to growth stunting carried out on adolescent girls as much as 55.66%.

**Table 6:** Results of the perception questionnaire answers per question

Indicator	Strongly agree		Agree		Neutral		Disagree		Strongly disagree	
	*n*	%	*n*	%	*n*	%	*n*	%	*n*	%
**Awareness**										
1.The important role of dentists	73	68.87	32	30.19	1	0.94	0	0.00	0	0.00
2. Contribution of 1000 HPK dentists	74	69.81	30	28.30	2	1.89	0	0.00	0	0.00
3. Dentist detects growth stunting	59	55.66	39	36.79	5	4.72	3	2.83	0	0.00
4. Dentists take care of oral health	77	72.64	26	24.53	2	1.89	1	0.94	0	0.00
13. The relationship between growth stunting and the oral health of mothers and children	63	59.43	41	38.68	1	0.94	1	0.94	0	0.00
14. The relationship between growth stunting and children's oral health	58	54.72	42	39.62	5	4.72	1	0.94	0	0.00
**Adoption**										
5. Dentists contribute to ANC	58	54.72	35	33.02	9	8.49	4	3.77	0	0.00
6. Dentist intervention 1000 HPK	62	58.49	37	34.91	6	5.66	1	0.94	0	0.00
7. Dentists help early detection of disease	65	61.32	35	33.02	5	4.72	1	0.94	0	0.00
9. Dentist education diet	70	66.04	35	33.02	1	0.94	0	0.00	0	0.00
12. Oral healthy behavior	63	59.43	42	39.62	1	0.94	0	0.00	0	0.00
**Implementation**										
11. Dentists play an active role in preventing growth stunting	62	58.49	41	38.68	3	2.83	0	0.00	0	0.00
16. ECC prevention and growth stunting support each other	66	62.26	37	34.91	1	0.94	2	1.89	0	0.00
17. Dentists play a role in maintaining the oral health of pregnant women	67	63.21	38	35.85	1	0.94	0	0.00	0	0.00
**Maintenance**										
8. Prevention of growth stunting for various categories	65	61.32	33	31.13	7	6.60	1	0.94	0	0.00
10. Health education and growth stunting are carried out for adolescent girls	59	55.66	39	36.79	7	6.60	1	0.94	0	0.00
15. Education on oral health behavior is given to the bride and groom	61	57.55	41	38.68	4	3.77	0	0.00	0	0.00
18. Nutritional advice by dentist is important	60	56.60	43	40.57	3	2.83	0	0.00	0	0.00
19.Active promotion of healthy food	70	66.04	35	33.02	1	0.94	0	0.00	0	0.00
20. Growth stunting can be easily prevented: clean and healthy living behavior	61	57.55	40	37.74	5	4.72	0	0.00	0	0.00

Abbreviations: ANC, ante natal care; ECC, early childhood caries; HPK, (hari pertama kehidupan = first day of life).

**Table 7:** Dentists' perceptions of growth stunting and its prevention in Bandung

Perception	*n*	%
Positive	57	53.77
Negative	49	46.23
Total	106	100.00

The results of obtaining participants' answers regarding the perception of growth stunting and its prevention are divided into positive and negative perceptions. From the analysis results, the total mean perception of dentists in the city of Bandung is 91.24, so the perception is said to be positive if the score is 91.24, and it is said to be negative if the score is less than 91.24. Dentists' perceptions of growth stunting and its prevention in the city of Bandung can be seen in Table 5 . Positive and negative perceptions regarding growth stunting and its prevention among dentists in Bandung are almost balanced at 53.77 versus 46.23%.

## Discussion

Short nutritional status, commonly known as growth stunting, is a form of undernutrition measured based on 2005 WHO reference standard deviation. 20 Short nutritional status (growth stunting) is when a person's height is shorter than the height of other people his age. A child is categorized as growth stunting when his height is less than −2 SD WHO Child Growth Standards median. 21 This is caused by one of the conditions that causes a person to experience chronic nutritional deficiencies that last for a long time, such as poverty, unhealthy living behavior, and poor parenting or eating patterns since the child is born. Malnutrition occurs in intrauterine and the early days of birth, but these symptoms appear when the child is 2 years old. 11

Growth stunting is a condition of growth failure in children caused by long-term malnutrition. Inadequate energy and protein consumption can affect tooth size and enamel solubility, salivary gland hypofunction, and inhibit tooth eruption. Meanwhile, vitamin D and A deficiency and protein-energy malnutrition (PEM) are associated with enamel hypoplasia. Salivary gland hypofunction related to PEM causes a decrease in salivary flow rate, buffer capacity, and a decrease in the composition of saliva, especially protein. 14 Growth stunting can also cause dental and oral health problems that are very common in children. Dental caries is the most common oral health problem in stunted growth children with impaired salivary protection function associated with reduced salivary flow rate and oral cleansing by antibacterial proteins. Because saliva flow decreases and then reduces salivary buffer and self-cleansing, which can ultimately increase the risk of dental caries. 20

The Government of Indonesia has determined the role of dentists and plans for preventing growth stunting in various activities to reduce the incidence of growth stunting in PERMENKES RI No. 39 of 2016. The efforts are aimed at various categories: pregnant women, toddlers, school children, adolescents, and young adults. There are several things dentists can do to reduce the number of growth stunting in Indonesia, such as contributing to ANC, the first 1,000 days of intervention, and assisting in the early detection of disease. 1 Study results showed that the knowledge of dentists in the city of Bandung regarding growth stunting and its prevention was included in the category of good knowledge, and the perception of dentists in the city of Bandung on growth stunting and its prevention was positive. Therefore, based on these results, dentists in the Bandung should have sufficient knowledge and a good perception of growth stunting to prevent growth stunting.

The item that received the highest total score is the one regarding the definition of growth stunting, which proved that dentists' knowledge has been excellent in relevant to the study by Sutarto. 21 The item with the lowest score in the knowledge section is the growth stunting question related to the mother's oral cavity health condition. This result shows that knowledge about it is still lacking. Based on study by Patera Nugraha et al, the oral cavity is the first entry point for food into the body before it is further processed in the gastrointestinal tract; therefore, poor oral health will cause a decrease in nutrient absorption and can cause the mother to be malnourished so that it can affect the fetus. In addition, diseases of the oral cavity, one of which is periodontitis, can cause fetal problems such as preeclampsia. 1

Statements with answers that strongly agree are primarily found in items about dentists who have played an essential role in maintaining dental and oral health as the main route of entry of nutrients into the body. The statement with the lowest perception score is about the dentist contributing to the implementation of ANC. As a dentist, there are several things that they can do to take part in reducing the number of growth stunting in Indonesia, such as participating in contributing to ANC, the first 1,000 days of intervention, and assisting in the early detection of disease. 1

So far, the authors have not found any research on dentists' knowledge and perceptions of growth stunting. However, a similar study has been conducted by Kamal Elden et al on groups of health workers at primary health care centers in Giza Province, Egypt. 22 The results of this study follow the results of research that have been carried out at this time, namely the majority of health workers already have good knowledge about growth stunting. However, this study differs from the results of research by Liem et al, which states that although the term growth stunting is increasingly known, it has not been accompanied by a strong perception. 23

This study obtained promising results because most dentists in the city of Bandung have good knowledge and perceptions about growth stunting and its prevention. This result can be used as a reference for further research on the efforts made by dentists to prevent growth stunting as a follow-up to dentists who already have good knowledge and perception is also recommended. Suggestions for the government and the involvement of dentists in the growth stunting prevention program need to be increased and clarified to help reduce the growth stunting rate in the city of Bandung and also in Indonesia. Although it is promising, there are limitations to this study. For example, limitations in reaching a larger population which involves various levels of cities in Indonesia so that it can genuinely answer the representation of dentists as a population in whole.

## Conclusion

Most dentists in Bandung City have a good level of knowledge about growth stunting and its prevention. However, socialization regarding the role of dentists in growth stunting prevention needs to be intensified so that the knowledge and perceptions possessed by these dentists can be implemented into actual actions in the field.
